# Phase-Field Simulation of Spinodal Decomposition in U-50Zr Metallic Nuclear Fuel

**DOI:** 10.3390/nano14191548

**Published:** 2024-09-25

**Authors:** Yongxiao La, Chunyang Wen, Linna Feng, Yihui Luo, Di Yun, Wenbo Liu

**Affiliations:** 1 Department of Nuclear Science and Technology, Xi’an Jiaotong University, Xi’an 710049, China; lyx3201691339@stu.xjtu.edu.cn (Y.L.); fenglinna@stu.xjtu.edu.cn (L.F.); 1263391582@stu.xjtu.edu.cn (Y.L.); diyun1979@xjtu.edu.cn (D.Y.); 2 Department of Materials, School of Natural Sciences, The University of Manchester, Sackville Street, Manchester M13 9PL, UK; chunyang.wen@postgrad.manchester.ac.uk; 3 Shaanxi Key Laboratory of Advanced Nuclear Energy and Technology, Xi’an Jiaotong University, Xi’an 710049, China

**Keywords:** phase-field model, U-50Zr alloy, spinodal decomposition, grain boundary

## Abstract

During the γ phase–δ phase transition, U-50Zr fuel experiences spinodal decomposition, which has a significant effect on fuel properties. However, little is known about the spinodal decomposition of U-50Zr. The spinodal decomposition behavior in U-50Zr is studied in this research using the phase-field approach. The mechanism of spinodal decomposition from a thermodynamic perspective is studied, and the effects of temperature and grain boundary (GB) on spinodal decomposition are analyzed. It is found that the concentration of U atoms in the U-rich phase formed during spinodal decomposition is as high as 90%. The U-rich phase first appears at the GB position, and precipitation phases appear inside the grain later. Ostwald ripening occurs when larger precipitation phases on the GB absorb isolated smaller precipitation phases inside the grain. The coarsening rate of precipitation phases and the time it takes for spinodal decomposition to achieve equilibrium are both influenced by temperature.

## 1. Introduction

U-50Zr alloy fuel has numerous notable advantages over other metallic nuclear fuels [1,2,3,4], making it the primary nuclear fuel for the fourth-generation reactors [5,6]. However, experimental studies have shown that U-50Zr undergoes spinodal decomposition in the ω phase region during high-temperature annealing experiments [7,8]. There have been numerous studies on the phenomenon and process of spinodal decomposition [9,10,11], which is a type of homogeneous transformation that plays a unique and crucial role in many alloy materials. It is a significant solid-state phase transition process in modern materials science. While there is limited research on the appearance and brief existence of the ω phase during the transformation from the γ to δ phase in U-Zr alloys, there is a certain understanding of the mechanism of this martensitic transformation process [12,13].

Spinodal decomposition is the process of generating subphases with the same structure but with different components from the parent phase. Due to the fact that the occurrence domain of spinodal decomposition is usually at the nanoscale, and the structure evolution during the decomposition process is very rapid, early experimental research on spinodal decomposition is extremely difficult to observe. Nowadays, with the help of in situ transmission electron microscopy, atomic probe tomography, and other experimental observation methods, the microstructure and morphology of materials can be directly observed at the atomic scale. In 2020, Tian et al. [7] found that during high-temperature annealing experiments on U-50Zr, spinodal decomposition occurred in the ω phase region of the U-50Zr alloy. For the first time, thermal annealing-induced spinodal decomposition of U-50Zr alloy was observed at 620 °C. The spinodal decomposition phenomenon of U-50Zr alloy was also observed at 550 °C through ion irradiation at 1 MeV. Although spinodal decomposition has been observed in other alloy systems [14,15,16], it is worth noting that this is the first time that spinodal decomposition has been observed in U-Zr alloys. This is an important phenomenon that leads to significant changes in the physical properties of U-Zr alloys. In 2021, Tian et al. [8] successfully captured the spinodal decomposition of U-50Zr alloy with temperature and the subsequent transition to binary phase separation using in situ transmission electron microscopy. Through a series of heating experiments conducted between room temperature and 1000 °C, it was found that the temperature at which the U-50Zr hexagonal parent phase begins to undergo spinodal decomposition is 575 °C. U-50Zr was annealed at 600 °C for 30 min, and the nano-microstructure and element distribution were observed. Khanolkar et al. [17] monitored the microstructure evolution of polycrystalline binary U-Zr alloys, U-20Zr, U-50Zr, and U-80Zr during the heat treatment process using laser ultrasound. Through ultrasound measurement combined with in situ transmission electron microscopy observation, it was found that above 600 °C, the δ–UZr_2_ in all three components underwent spinodal decomposition, forming nanoscale regions.

The spinodal decomposition of U-50Zr will significantly impact the microstructure and service performance of the alloy. Therefore, studying the spinodal decomposition process of U-50Zr and simulating the microstructural evolution during this process holds considerable engineering and scientific value. This article employs the mesoscale phase-field method to simulate the spinodal decomposition of U-50Zr, utilizing thermodynamic free energy as the driving force to investigate the mechanism of spinodal decomposition from a thermodynamic perspective.

## 2. Phase-Field Method

**Figure 1:**
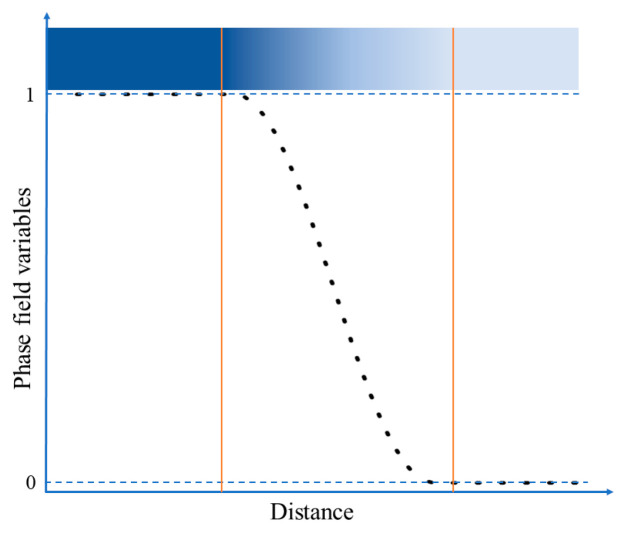
Schematic diagram of the diffusion interface for phase-field variables.

The phase-field model for spinodal decomposition has also been utilized in other metals [18,19,20,21]. In previous phase-field simulation studies, artificially constructed double potential well functions were often used as the driving force for spinodal decomposition processes. Although they can fully demonstrate the phase transition process, they lack a certain physical basis. This paper constructs the free energy expression of U-50Zr based on the sub-regular solute model of thermodynamic theory, which serves as the primary driving force for the spinodal decomposition. Considering that the concentration of Zr atoms in different phase regions will vary when U-50Zr undergoes spinodal decomposition, to describe the evolution of the system’s microstructure, it is essential to determine the changes in the Zr atomic concentration over time and space. Therefore, a conserved field variable, $c_{Zr}$, is introduced to represent the atomic concentration of Zr. The concentration of another group of fractional U atoms in U-Zr alloys can be represented by 1 − $c_{Zr}$. A set of non-conserved field variables, $\eta_{1}$, $\eta_{2}$, …, $\eta_{n}$ are also introduced to describe the grains with different orientations. Inside the *i*th grain, the field variable $\eta_{i}=1$, and all the other order parameters are 0. The grain orientation in actual materials is infinite, using a finite but large number of grain orientation numbers (e.g., *n* = 36) [22] can accurately simulate the grain evolution process, therefore, the selected grain orientation number in this article is *n* = 36. At the grain boundary (GB), the order parameters are continuously varied with a smooth transition, as shown in Figure 1.

### 2.1. Free Energy Function

The total free energy of the system includes the bulk free energy and the gradient free energy, and in thermodynamic theory, the bulk free energy refers to the total free energy of the ω phase. The expression for the total free energy of the system is given by the following equation [23]:

(1) $$F=\int_{V}\left\{\frac{1}{V_{m}}\left(f_{chem}+f_{\mathrm{int}er}\right)+\frac{1}{2}\kappa_{c_{Zr}}{\left(\nabla c_{Zr}\right)}^{2}+\sum_{i=1}^{n}\frac{1}{2}\kappa_{\eta }{\left(\nabla \eta_{i}\right)}^{2}\right\}dV$$

where $V_{m}$ is the reference molar volume of U-50Zr, $\kappa_{c_{Zr}}$ and $\kappa_{\eta }$ are gradient energy coefficients. According to the solute model, the free energy consists of the molar free energy of the ideal solute and the excess free energy, and it is shown as follows [23]:

(2) $$\begin{array}{l} f_{chem}=c_{Zr}G_{0}^{\omega Zr}+(1-c_{Zr})G_{0}^{\omega U}+c_{Zr}(1-c_{Zr})I_{c_{Zr}c_{U}}\\ \;\;\;\;\;\;\;\;\;\;\;+RT[c_{Zr}\ln c_{Zr}+(1-c_{Zr})\ln(1-c_{Zr})] \end{array}$$

where *R* represents the ideal gas constant, *T* is the temperature of the system, and $I_{c_{Zr}c_{U}}$ is the interaction energy of Zr and U.

$G_{0}^{\omega Zr}$ and $G_{0}^{\omega U}$ are the intrinsic free energies (unit of J/mol) of Zr and U in the ω phase, respectively. U is hexagonal (orthorhombic) in the ω phase, and Zr remains in the ω phase. Their intrinsic free energies are given by the following equations [24]:

(3) $$\begin{array}{l} G_{0}^{\omega Zr}=144.432234T-26.8556T\ln T-2.7994455e^{-3}T^{2}\\ \;\;\;\;\;\;\;\;\;\;\;\;\;+38376T^{-1}-8878.082+G_{pres} \end{array}$$

(4) $$\begin{array}{l} G_{0}^{\omega U}=130.9552T-26.9182T\ln T+1.2516e^{-3}T^{2}\\ \;\;\;\;\;\;\;\;\;\;\;\;\;-4.4261e^{-6}T^{3}+38568T^{-1}-8407.734 \end{array}$$

The interaction energy between the conserved variables and the non-conserved variables can be expressed as follows [25]:

(5) $$f_{\mathrm{int}er}=m(c_{Zr})\left[\frac{1}{4}+\sum_{i=1}^{n}\left(\frac{1}{4}\eta_{i}^{4}-\frac{1}{2}\eta_{i}^{2}\right)+\sum_{i=1}^{n}\sum_{j\neq i}^{n}\eta_{i}^{2}\eta_{j}^{2}\right]$$

where $m(c_{Zr})$ is component-related factor, and it is denoted as follows:

(6) $$m(c_{Zr})=1+Ac_{Zr}^{2}-Bc_{Zr}^{2}{\left(1-c_{Zr}\right)}^{2}$$

where *A* and *B* are constants, *A* = 0.6 and *B* = 6.0.

Due to the limitation of thermodynamic studies of the ω phase, there are no available interaction energy expressions. Therefore, in this work, an empirical expression for the interaction energy is proposed based on current experimental results.

In formal solute approximation models, the interaction energy is typically assumed to be constant, which does not accurately reflect the molar free energy of the solute. In this model, we will consider the interdependence of binding energy between atoms and temperature. The interaction energy will be treated as a primary function of temperature, providing a more precise description of the molar free energy of the solute. The interaction energy significantly influences the total free energy of the system and plays a crucial role in determining whether spinodal decomposition can occur. Once it reaches a specific threshold, the spinodal decomposition process becomes clearly observable in experiments. In this study, it was found that the destabilizing phase transition can be clearly observed within a specific time range only when the interaction energy reaches 20,250 J/mol. The temperature at which this occurs is 575 °C, which also represents the minimum temperature for spinodal decomposition to take place. As the temperature increases, the process of spinodal decomposition accelerates. By considering temperature as a primary factor, we can establish the ratio coefficient between interaction energy and temperature, based on the presence or absence of the spinodal decomposition process at various temperatures during the experiment. From the data presented, it is evident that the relationship between interaction energy $I_{c_{Zr}c_{U}}$ and temperature is $I_{c_{Zr}c_{U}}=23.875T$. Although this result may not be entirely precise, it is sufficient to solve many current problems. Especially considering the instability of the ω phase structure, the calculation based on first principles is also considered to be very impractical. Consequently, the relationship derived from the experimental data is considered to be acceptable.

### 2.2. Kinetic Equations

The phase transition process of spinodal decomposition is related to the diffusion of the component, and the Cahn–Hilliard equation has the ability to describe the evolution of component diffusion very well. The Cahn–Hilliard equation which can describe the evolution of Zr concentration with time is shown as the following equation [26]:

(7) $$\frac{\partial c_{Zr}}{\partial t}=\nabla \cdot \left(M_{Zr}\nabla \frac{\delta F}{\delta c_{Zr}}\right)$$

where $M_{Zr}$ is the chemic mobility coefficient of Zr atoms, and it is coupled by the atomic mobility coefficients of U and Zr in the ω phase, its expression is as follows [27]:

(8) $$M_{Zr}=\frac{V_{m}}{RT}c_{Zr}\left(1-c_{Zr}\right)(c_{Zr}D_{Zr}+\left(1-c_{Zr}\right)D_{U})$$

where $D_{Zr}$ and $D_{U}$ are the atomic mobility coefficients of Zr and U, respectively, which can be calculated by the following equations [28]:

(9) $$D_{U}=D_{0}^{U}e^{\frac{-H_{U}}{RT}},\;\;\;\;D_{Zr}=D_{0}^{Zr}e^{\frac{-H_{Zr}}{RT}}$$

where $D_{0}^{U}$ and $D_{0}^{Zr}$ are diffusion coefficient factors, $H_{U}$ and $H_{Zr}$ are the activation energies for the migration of U and Zr in the ω phase, respectively. 

Since the ω phase is located within the miscible gap in the phase diagram, which is unstable and lacks a fixed structure, determining the activation energy and other relevant parameters for atom migration in the ω phase is challenging. Currently, no one has calculated these parameters. However, it is evident that the diffusion rate of atoms in the ω phase is accelerated due to spinodal decomposition. Additionally, the ω phase and γ phase have some structural similarities; therefore, in this study, we propose approximating the migration coefficients of the ω phase using the atomic migration coefficient value of the γ phase. 

The evolution of non-conserved field variables can be described by the following Allen–Cahn equation [29]:

(10) $$\frac{\partial \eta_{i}}{\partial t}=-L\frac{\delta F}{\delta \eta_{i}}$$

where *L* is the phase mobility. 

### 2.3. Equation Solving

In this paper, semi-implicit Fourier prime method is used to solve the evolution equations. The Fourier transform is applied to both sides of Equation (7) and the space is discretized as follows:

(11) $$\frac{\partial {\left\{c_{Zr}\right\}}_{k}}{\partial t}=-k^{2}M_{Zr}\left[{\left\{\frac{1}{V_{m}}\frac{\delta \left(f_{chem}+f_{\mathrm{int}er}\right)}{\delta c_{Zr}}\right\}}_{k}+k^{2}\kappa_{c_{Zr}}{\left\{c_{Zr}\right\}}_{k}\right]$$

where ${\left\{\cdot \right\}}_{k}$ is the Fourier transform of the variable in parentheses, k is a vector in Fourier space, $k=\left(k_{1},k_{2}\right)$, and the order of magnitude is $\sqrt{k_{1}^{2}{+k}_{2}^{2}}$.

Expanding the right hand side of Equation (11) yields the following:

(12) $$\frac{\partial {\left\{c_{Zr}\right\}}_{k}}{\partial t}=-k^{2}M_{Zr}{\left\{\frac{1}{V_{m}}\frac{\delta \left(f_{chem}+f_{\mathrm{int}er}\right)}{\delta c_{Zr}}\right\}}_{k}-k^{4}M_{Zr}\kappa_{c_{Zr}}{\left\{c_{Zr}\right\}}_{k}$$

By implicitly dealing with linear terms and fourth-order operators and explicitly dealing with nonlinear terms, the semi-implicit form of Equation (12) can be written as follows:

(13) $$\frac{{\left\{c_{Zr}\right\}}_{k}^{n+1}-{\left\{c_{Zr}\right\}}_{k}^{n}}{\Delta t}=-k^{2}M_{Zr}{\left\{\frac{1}{V_{m}}\frac{\delta \left(f_{chem}+f_{\mathrm{int}er}\right)}{\delta c_{Zr}}\right\}}_{k}^{n}-k^{4}{Μ}_{Zr}\kappa_{c_{Zr}}{\left\{c_{Zr}\right\}}_{k}^{n+1}$$

where Δ*t* is represents interval of time with the value of 0.01 in this article.

By organizing Equation (13), the following can be concluded:

(14) $${\left\{c_{Zr}\right\}}_{k}^{n+1}=\frac{{\left\{c_{Zr}\right\}}_{k}^{n}-\Delta tk^{2}M_{Zr}{\left\{\frac{1}{V_{m}}\frac{\delta \left(f_{chem}+f_{\mathrm{int}er}\right)}{\delta c_{Zr}}\right\}}_{k}^{n}}{1+\Delta tk^{4}M_{Zr}\kappa_{c_{Zr}}}$$

After solving the above discrete equations, a Fourier inverse transform is performed to obtain the actual concentration values. 

Similarly, the Fourier transform is applied to both sides of Equation (10):

(15) $$\frac{\partial {\left\{\eta_{i}\right\}}_{k}}{\partial t}=-L{\left\{\frac{1}{V_{m}}\frac{\delta \left(f_{chem}+f_{\mathrm{int}er}\right)}{\delta \eta_{i}}\right\}}_{k}-k^{2}L\kappa_{\eta }{\left\{\eta_{i}\right\}}_{k}$$

The semi-implicit form of Equation (15) can be written by implicitly dealing with linear terms and fourth-order operators and explicitly dealing with nonlinear terms, as follows:

(16) $$\frac{{\left\{\eta_{i}\right\}}_{k}^{n+1}-{\left\{\eta_{i}\right\}}_{k}^{n}}{\Delta t}=-L{\left\{\frac{1}{V_{m}}\frac{\delta \left(f_{chem}+f_{\mathrm{int}er}\right)}{\delta \eta_{i}}\right\}}_{k}^{n}-k^{2}L\kappa_{\eta }{\left\{\eta_{i}\right\}}_{k}^{n+1}$$

Organizing the above equation gives the following:

(17) $${\left\{\eta_{i}\right\}}_{k}^{n+1}=\frac{{\left\{\eta_{i}\right\}}_{k}^{n}-\Delta tL{\left\{\frac{1}{V_{m}}\frac{\delta \left(f_{chem}+f_{\mathrm{int}er}\right)}{\delta \eta_{i}}\right\}}_{k}^{n}}{1+\Delta tLk^{2}\kappa_{\eta }}$$

Solving the above discrete equations and then performing the Fourier inverse transform can yield the values of the order parameters in real space.

### 2.4. Reduced Dimensionless Variables 

In this paper, the explicit Euler algorithm of finite difference is used to solve the Cahn–Hilliard and Allen–Cahn equations, and the evolution equations need to be dimensionless when programming for solution. In order to briefly explain the dimensionless process, evolution equations are selected as representatives, as follows:

(18) $$\frac{\partial c_{Zr}}{\partial t}=\nabla M_{Zr}\nabla \left(\frac{1}{V_{m}}\frac{\delta \left(f_{chem}+f_{\mathrm{int}er}\right)}{\delta c_{Zr}}-\kappa_{c_{Zr}}\nabla^{2}c_{Zr}\right)$$

(19) $$\frac{\partial \eta_{i}}{\partial t}=-L\left(\frac{1}{V_{m}}\frac{\delta \left(f_{chem}+f_{\mathrm{int}er}\right)}{\delta \eta_{i}}-\kappa_{\eta }\nabla^{2}\eta_{i}\right)$$

Here, a characteristic length l* with the unit of a meter and a characteristic time t* with the unit of a second are used to make the length and time dimensionless by the definition of *λ* = *l*/l* and *τ* = *t*/t*, where *l* and *t* are actual length and time, respectively. The dimensionless transformation of gradient is $\tilde{\nabla }=l^{*}\nabla$, and the units of *M_Zr_*, *f*, *κ* are m^5^/(J·s), J, and J/m, respectively. Therefore, we introduce a characteristic energy density e* (J/m^3^) to make the Cahn–Hilliard and Allen–Cahn equations dimensionless, as follows:

(20) $$\begin{array}{l} \frac{\partial c_{Zr}}{\partial \tau }=\frac{t^{*}}{l^{*}}\tilde{\nabla }M_{Zr}e^{*}\tilde{\nabla }\frac{1}{l^{*}}\left(\frac{1}{V_{m}}\frac{\delta \left(\left(f_{chem}+f_{\mathrm{int}er}\right)/e^{*}\right)}{\delta c_{Zr}}-\frac{\kappa_{c_{Zr}}}{e^{*}{\left(l^{*}\right)}^{2}}{\tilde{\nabla }}^{2}c_{Zr}\right)\\ \;\;\;\;\;=\tilde{\nabla }\left({\tilde{M}}_{Zr}\tilde{\nabla }\left(\frac{\delta \left({\tilde{f}}_{chem}+{\tilde{f}}_{\mathrm{int}er}\right)}{\delta c_{Zr}}-{\tilde{\kappa }}_{Zr}{\tilde{\nabla }}^{2}c_{Zr}\right)\right) \end{array}$$

(21) $$\begin{array}{l} \frac{\partial \eta_{i}}{\partial \tau }=-t^{*}e^{*}L\left(\frac{\delta \left(\left(f_{chem}+f_{\mathrm{int}er}\right)/e^{*}\right)}{\delta \eta_{i}}-\frac{\kappa_{\eta }}{e^{*}{\left(l^{*}\right)}^{2}}{\tilde{\nabla }}^{2}\eta_{i}\right)\\ \;\;\;\;\;=-\tilde{L}\left(\frac{\delta \left({\tilde{f}}_{chem}+{\tilde{f}}_{\mathrm{int}er}\right)}{\delta \eta_{i}}-{\tilde{\kappa }}_{\eta }{\tilde{\nabla }}^{2}\eta_{i}\right) \end{array}$$

Therefore, the dimensionless parameters are $\tilde{M}=Mt^{*}e^{*}/{\left(l^{*}\right)}^{2}$, $\tilde{f}=f/e^{*}$, $\tilde{\kappa }=\kappa /\left(e^{*}{\left(l^{*}\right)}^{2}\right)$, and $\tilde{L}=Lt^{*}e^{*}$.

The selection principle of characteristic values is to make the magnitude of dimensionless parameters close to each other, and a set of characteristic values in this paper are as follows:

(22) $$\left\{\begin{array}{l} e^{*}=RT/V_{m}\\ t^{*}=\frac{1}{Le^{*}}\\ l^{*}=\sqrt{\kappa_{\eta }/e^{*}} \end{array}\right.$$

In the present work, the phase-field model is applied to simulate the spinodal decomposition of U-50Zr alloy in both two-dimensional (2D) and three-dimensional (3D) simulations. 

## 3. Results

### 3.1. Spinodal Decomposition within Grain of U-50Zr

The intracrystalline spinodal decomposition of U-50Zr is studied in this section. For simplicity, the phase-field model of intracrystalline spinodal decomposition does not need to introduce the evolution of non-conserved field variables, and only the evolution of Zr atom concentration needs to be solved. According to the study of energy functional theory and first principles, it is predicted that mixed-phase gaps will occur in the U-Zr alloy system when the composition range of Zr is within specific limits. Moreover, experimental observations have demonstrated that the U-50Zr alloy undergoes spinodal decomposition within this phase region. According to experimental observations, the minimum temperature at which the U-50Zr alloy begins spinodal decomposition without irradiation is 575 °C [8]. The phase-field model presented in this section was developed based on the actual thermodynamic parameters of the U-50Zr alloy. The simulation parameters for this section are detailed in Table 1, with a 2D simulation area size of 128 × 128.

**Figure 2:**
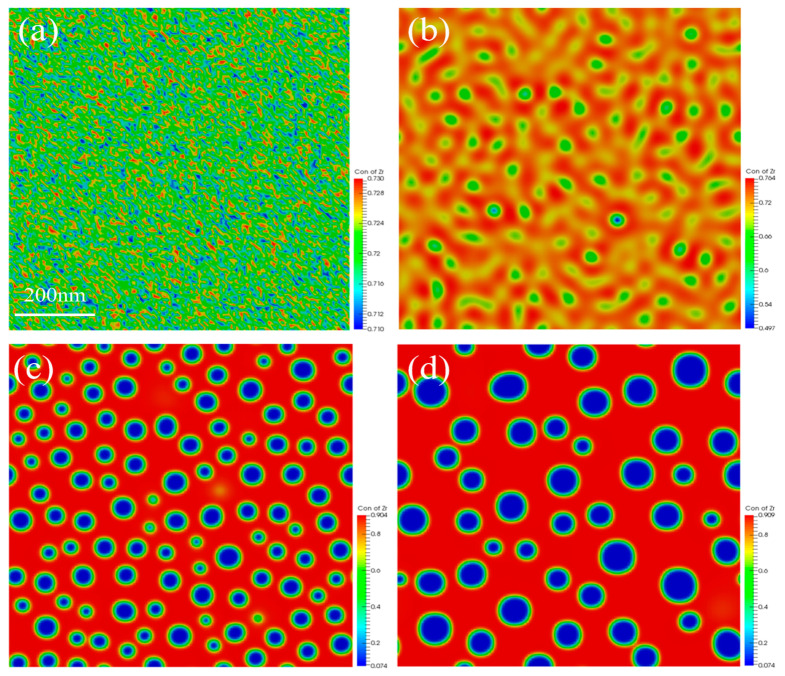
Spinodal decomposition at 973 K. (**a**) 0 s, (**b**) 300 s, (**c**) 1500 s, and (**d**) 9000 s.

The U-50Zr spinodal decomposition process at a temperature of 700 °C was firstly simulated in a simulation area measuring 900 nm × 900 nm, with periodic boundary conditions adopted. Initially, the concentration of Zr was distributed relatively uniformly, with only noise-induced fluctuations serving as an initial perturbation. In the spinodal regime, alloys are inherently susceptible to infinitesimal compositional fluctuations introduced by external stimuli, such as thermal aging [35], mechanical strain [36], and ionizing or displacive irradiation [37]. The simulation results are shown in Figure 2, which illustrates the initial and final states of the spinodal decomposition process, as well as the intermediate stages.

Observation and analysis of Figure 2 indicate that the concentration of Zr is initially distributed more uniformly, with only noise-induced concentration fluctuations as the initial perturbation of the spinodal decomposition, as illustrated in Figure 2a. As the spinodal decomposition progresses, the U-rich phase precipitates slowly, which aligns with the experimental results. However, the initial amount of precipitated U and Zr is insufficient, resulting in a structure that exhibits a worm-like shape, as shown in Figure 2b. Over time, the quantity of precipitation gradually increases, and the precipitated structure begins to coarsen, transitioning from a worm-like shape to a spherical shape. The spherical structures continue to grow, as depicted in Figure 2c,d.

**Figure 3:**
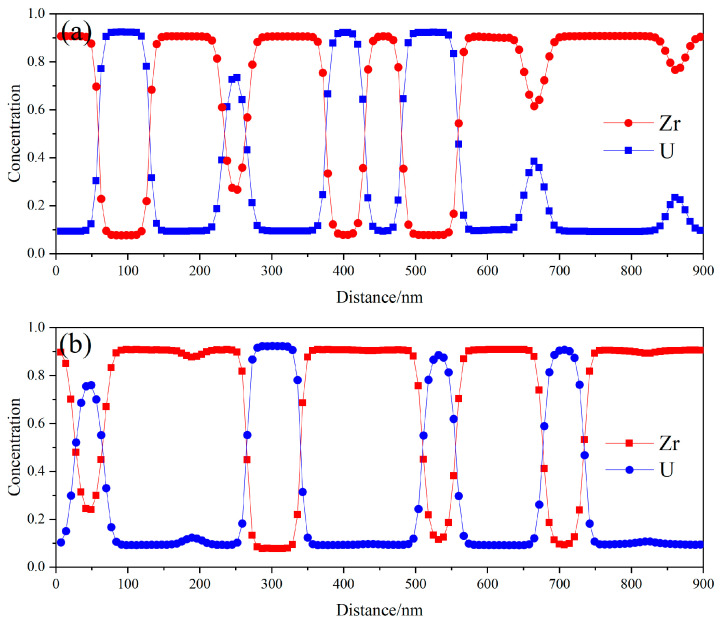
Elemental distribution of Zr and U at (**a**) *x* = 450 nm and (**b**) *y* = 450 nm.

The precipitation of phases results in a change in the distribution of the compositions, with the sum of the atomic concentrations of Zr and U totaling 100%. At the initial time, the average concentration of Zr atoms is 72%, with a fluctuation of 2%, as stated in this paper. As spinodal decomposition progresses, the concentration of Zr atoms in the precipitated U-rich phase gradually decreases, while the concentration of Zr atoms in the matrix phase gradually increases. Once spinodal decomposition reaches a sufficient point, the concentration of Zr atoms in both the U-rich phase and the matrix phase stabilizes within a range of approximately 10–90%. Figure 3 illustrates the distribution of Zr and U atomic concentrations along the lines x = 450 nm and y = 450 nm during the final stage of spinodal decomposition. It is evident that the concentration of Zr in the matrix is approximately 90%, while the concentration of Zr in the U-rich phase decreases to about 10% [8]. It is noteworthy that the U-rich phase contains about 10% Zr when spinodal decomposition is fully completed, and the simulation results presented in this paper align well with the experimental findings.

**Figure 4:**
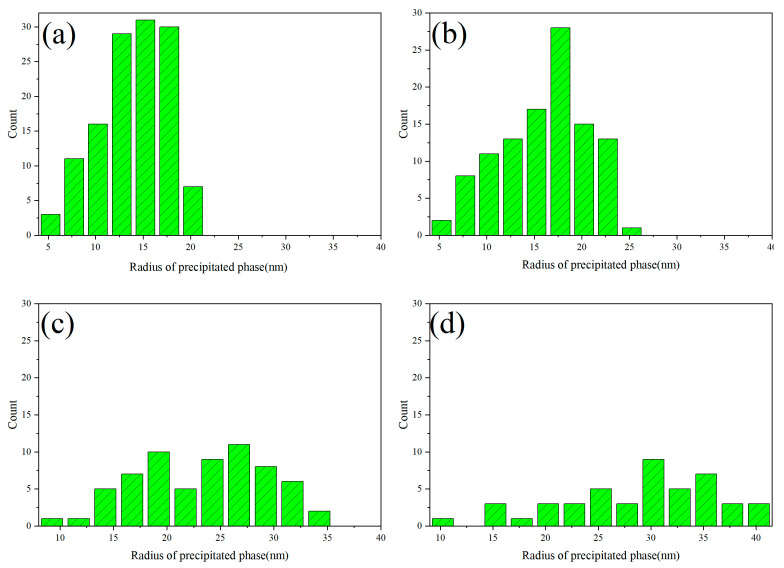
Radius distribution of precipitated phases. (**a**) 750 s, (**b**) 1500 s, (**c**) 4500 s, and (**d**) 9000 s.

In the process of spinodal decomposition, the precipitated phase gradually evolves from an initial worm-like structure to a spherical form. The spherical precipitation further evolves and grows by aggregating with other spherical forms, leading to the ripening process. The radius distribution of the precipitation during the aggregation and growth is statistically calculated in this part, and the results are presented in Figure 4.

When the shape of the precipitate transitions from worm-like to regularly spherical, measuring the radius of the precipitated phase becomes more straightforward. Figure 4 illustrates the change in the radius of the U-rich phase precipitates during the process of spinodal decomposition. The radius distribution of the precipitates during the initial period, when they transition from worm-like to spherical, is described in Figure 4a,b. These figures demonstrate that the radius distribution exhibits a uniform, single-peaked pattern, and fitting the radius distribution reveals that it follows a Gaussian distribution. As spinodal decomposition progresses, the spherical precipitates undergo aggregation and growth. The simulation results presented in this paper, in conjunction with experimental observations, indicate that the aggregation and growth of the U-50Zr spinodal decomposition precipitates obey the Ostwald ripening process. This process involves smaller precipitates dissolving into the matrix, with the dissolved atoms migrating into the larger precipitates to promote their growth. Consequently, the radius distribution of the precipitates becomes less uniform as spinodal decomposition advances to a specific stage, as illustrated in Figure 4c,d, where both small and large precipitates are present.

Through the research mentioned above, it was determined that the δ-UZr_2+x_ solid solution in the hexagonal phase begins to decompose at 575 °C. During this process, the parent phase decomposes into two sub-phases with the same structure but different compositions, namely U-rich and Zr-rich hexagonal phases, respectively. Initially, the concentration distribution is uniform, however, over time, the two phases begin to separate. Due to the higher initial concentration of Zr, the Zr-rich phases coalesce to form a Zr matrix, which facilitates the precipitation of the U phase. The initially structure of U phase is a worm-like shape, which subsequently connects and evolves into a spherical shape via slow coarsening.

### 3.2. Effect of Temperature on Spinodal Decomposition

**Figure 5:**
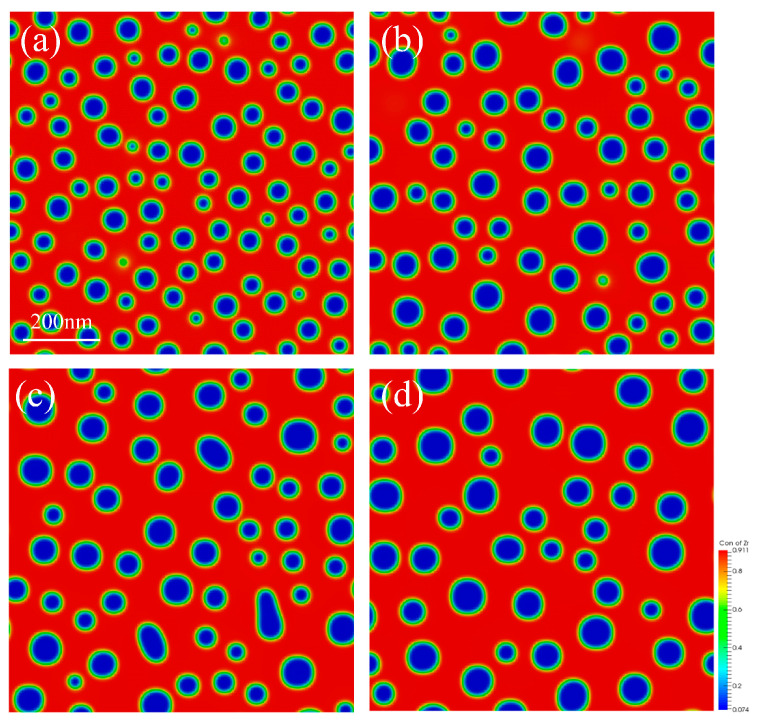
Morphology of spinodal decomposition at different temperatures: (**a**) 640 °C, (**b**) 670 °C, (**c**) 700 °C, and (**d**) 720 °C.

Experiments have demonstrated that spinodal decomposition is significantly influenced by temperature. Temperature not only determines the occurrence of spinodal decomposition but also affects the dynamics of the process. To investigate the effect of temperature on spinodal decomposition, various temperature conditions were established in this study. It is assumed that the temperature remains constant throughout spinodal decomposition. Figure 5 illustrates the U-50Zr intracrystalline spinodal decomposition at different temperatures. The simulation duration for spinodal decomposition is consistent across the various temperature settings. Therefore, the results presented in Figure 5 can qualitatively describe the effect of temperature on spinodal decomposition.

Through the observation and analysis of Figure 5, it can be observed that as the temperature increases, the spinodal decomposition rate of the U-50Zr alloy accelerates. This results in a shorter time to reach a phase equilibrium and an increased rate of structural coarsening. The influence of temperature on spinodal decomposition involves two key aspects. First, the process is closely linked to the migration and diffusion of elements within the U-Zr alloy, and the diffusion coefficients of these elements are temperature-dependent. Specifically, higher temperatures lead to larger diffusion coefficients [38,39], which facilitate spinodal decomposition. Additionally, the system’s free energy is also temperature-dependent; higher temperatures result in increased free energy, thereby accelerating the spinodal decomposition process. Consequently, raising the temperature is beneficial for spinodal decomposition.

**Figure 6:**
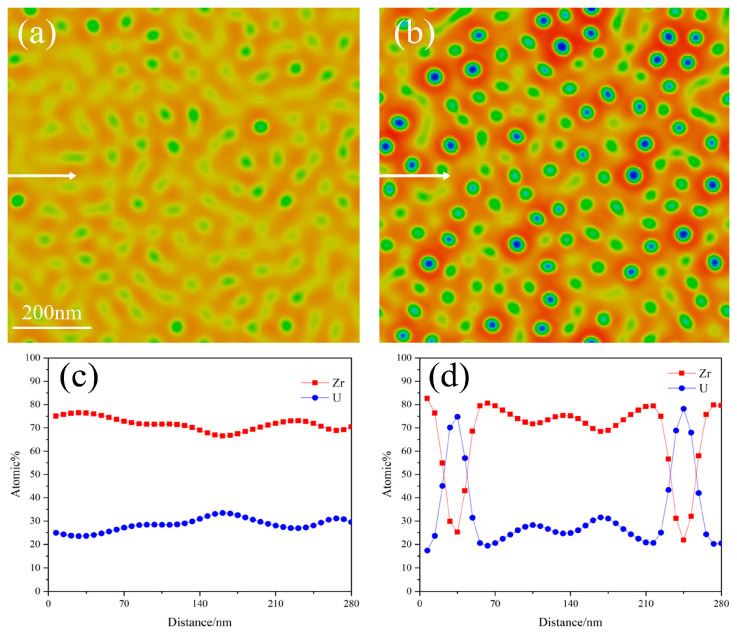
Phase separation of spinodal decomposition at different temperatures: (**a**) 640 °C and (**b**) 700 °C; (**c**,**d**) represent the distribution of elements along arrows.

Figure 6 illustrates the phase separation and element distribution along the direction of the arrow during the spinodal decomposition of a U-50Zr single crystal. It is evident that as the temperature increases, phase coarsening and phase separation occur more rapidly, resulting in a precipitate phase with a higher concentration of the U element. At 640 °C, the precipitate phase exhibits a worm-like morphology, with the concentration of U atoms in the precipitate phase not exceeding 30%. At this temperature, the precipitate phase has not yet fully separated from the parent phase. As the temperature reaches 700 °C, the precipitate phase displays both worm-like and spherical shapes, with the atomic concentration of U in the precipitate phase around 70%. Although the precipitate phase is still not completely separated at this temperature, a clear phenomenon of spinodal decomposition is already evident.

### 3.3. Effect of Grain Boundaries on Spinodal Decomposition

**Figure 7:**
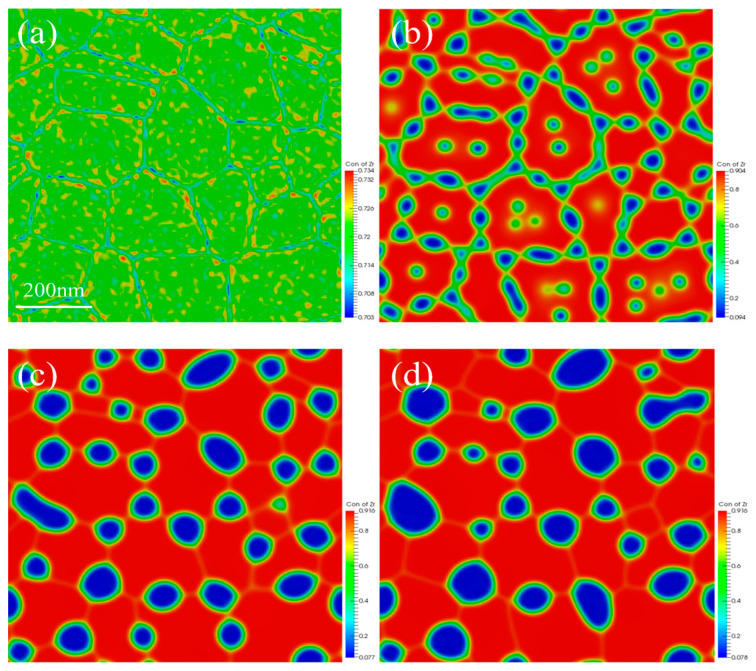
Spinodal decomposition in polycrystalline structures: (**a**) 0 s, (**b**) 375 s, (**c**) 3750 s, and (**d**) 7500 s.

Many factors can influence the progress of spinodal decomposition, including irradiation and defects. GBs, as a common type of defect, can also affect the spinodal decomposition. Therefore, it is essential to investigate the influence of GBs on the spinodal decomposition of U-50Zr. This section establishes a polycrystalline structure comprising 36 grains with different orientations, with a simulation temperature of 973 K and a simulation area measuring 900 nm × 900 nm. Periodic boundary conditions were applied. The initial Zr concentration is 72%, with component fluctuations reaching a maximum of 2%. In the polycrystalline spinodal decomposition model, it is crucial to introduce order parameters that characterize grain orientations, which will evolve over time. The specific model and dimensionless process are detailed in the model section. The simulation results are shown in Figure 7.

The simulation results indicate that the U-phase preferentially precipitates at GBs, as illustrated in Figure 7b. Over time, isolated U-rich phases also precipitate within the Zr matrix inside the grains. The size of the U-rich phases exhibits a bimodal distribution, consisting of larger, irregular U-rich phases located at the GBs and smaller, isolated U-rich phases located within the grains. As the evolution progresses, the larger U-phases at the GBs grow by absorbing the smaller U-phases within the grains. The microstructural evolution during coarsening is driven by traditional curvature coarsening, which aligns well with the phenomena observed in the experiments. Additionally, it is also evident that during the spinodal decomposition process, there is minimal grain growth. Although the initial grain size varies slightly as the spinodal decomposition proceeds, grain growth is not significant, and the positions of GBs remain unchanged. This stability is attributed to the U-rich precipitate phases formed during spinodal decomposition at the GBs, which create curvature that hinders the movement of the GBs and can even completely pin them in place.

Experimental observations have demonstrated that larger, irregular U-rich phases tend to aggregate near GBs, while smaller, spherical U-rich phases appear within the grains. As spinodal decomposition progresses, the larger U-rich phases located at the GB will undergo Ostwald ripening, a process in which the phases grow by absorbing smaller, isolated U-rich phases within the grain. Consequently, the number density of U-rich phases inside the grain will be significantly reduced, while the size of the U-rich phases at the GB will increase. This phenomenon is characteristic of typical coarsening process [40,41]. Polycrystalline spinodal decomposition is influenced by GBs, primarily due to the high atomic diffusion coefficient near the GBs [18,42,43]. Additionally, U-rich precipitation phases first appear at the GB positions, while isolated spherical U-rich phases precipitate within the Zr matrix of the grains.

### 3.4. Three-Dimensional Spinodal Decomposition

**Figure 8:**
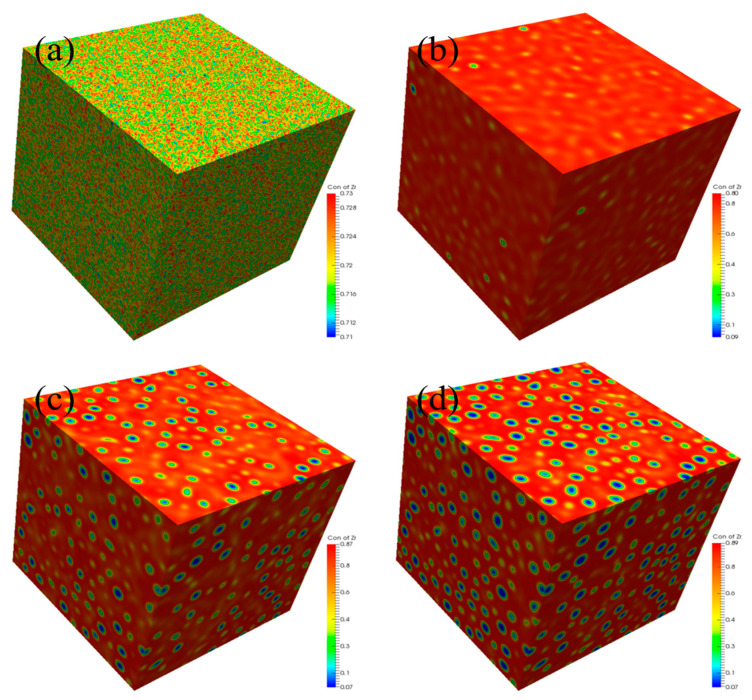
Three dimensional spinodal decomposition: (**a**) 0 s, (**b**) 600 s, (**c**) 720 s, and (**d**) 780 s.

In this part, the three-dimensional (3D) spinodal decomposition of U-50Zr is simulated. The simulation temperature is 973K, and the simulation area is 900 nm × 900 nm × 900 nm, with periodic boundary conditions adopted. The initial distribution of Zr is uniformly distributed, with 2% compositional fluctuations. The simulation results are shown in Figure 8. It can be seen that, in spinodal decomposition, the migration and aggregation of U atoms form a U-rich phase, whose shape gradually changes from worm-like to spherical, and the phase in the matrix is a Zr-rich phase. In the 3D simulation, the precipitated phase exhibited a non-spherical microstructure shape, which was influenced by nearby precipitated phases. However, this phenomenon was not observed in the 2D simulation. This is because the voxel-based microstructure of the 2D simulation comprised the four nearest neighboring voxels for the voxel allocated to the U-rich phase, whereas the 3D simulation comprised six [44].

It is accepted that sometimes a 2D-approach simulation is not a good proxy for a 3D system in physics (e.g., the 2D Ising vs. 3D Ising model for spin-lattice physics) [45]. During the spinodal decomposition of polycrystalline, the interaction between Zr atoms and GBs is coupled into the model using Equation (5). Consequently, the energy at the GB is lower than that inside the grains [46], making it more prone to form a U-rich phase near the GB (Figure 7). However, in the 3D simulation, the curvature of the spherical precipitate phase in the 3D simulation is too large, making it difficult for the precipitate phase to maintain its spherical shape [47]. As a result, the density of the U-rich phase at the GB in 3D simulation may be higher than that in 2D simulation, and the mutual influence between the precipitate phases may lead to the precipitation being bound together. Therefore, if the model developed in this study is applied to simulate the spinodal decomposition of polycrystalline U-50Zr alloy in 3D, many other factors, such as the interaction between solute atoms and GBs, the diffusion of solute atoms along GBs, and the distribution and morphology of GBs, should be considered. However, combining 2D simulation with 3D simulation is a new trend in phase-field research [48,49].

## 4. Conclusions

Based on the actual thermodynamic molar free energy, a phase-field method was established to study the spinodal decomposition of U-50Zr alloy in this article. The study involves simulating the evolution process of spinodal decomposition and analyzing the effects of temperature and grain boundaries (GBs) on spinodal decomposition. The main conclusions are as follows:

(1) There are slight composition fluctuations in the U-50Zr alloy as the initial condition of spinodal decomposition. During spinodal decomposition, U atoms in the alloy gradually precipitate and aggregate into U-rich phases. Over time, the precipitated elements begin to coarsen, and the structure evolves from worm-like to spherical. Simultaneously, the precipitate phase continues to grow through the Ostwald ripening mechanism.
(2) The model developed in this study is suitable for the simulation of spinodal decomposition of single crystal U-50Zr alloy in both two-dimensional (2D) and three-dimensional (3D) simulation. The simulation results showed that the temperature has a significant influence on the spinodal decomposition of U-50Zr. As the temperature increases, the time for the spinodal decomposition to reach equilibrium shortens, the rate of phase evolution accelerates, and the rate of coarsening increases. Research has found that temperature mainly affects spinodal decomposition by influencing the atomic diffusion coefficient and the Gibbs free energy of the system.
(3) The 2D simulated results about the spinodal decomposition of polycrystalline U-50Zr alloy showed that GBs have a significant impact on spinodal decomposition. The precipitation phase of spinodal decomposition initially occurs at the GBs, and isolated small spherical U-phases appear within the Zr matrix inside the grains later. As time progresses, large U-rich phases at GBs undergo Ostwald ripening, and grow by absorbing U atoms from the U-rich phase within the grains.
(4) The three-dimensional (3D) spinodal decomposition of U-50Zr single crystal was also simulated, and the simulation results showed that the phases after spinodal decomposition were isolated U-rich phase and matrix Zr-rich phase, respectively. The U-rich phase gradually evolved from an initial worm-like shape to a spherical shape. Due to the influence of surrounding U-rich phases, non-spherical U-rich phases may appear in 3D simulations. However, when the model developed in this study is applied to simulate the 3D spinodal decomposition of polycrystalline alloy, some other factors should be considered.

## Figures and Tables

**Table 1:** Spinodal decomposition simulation parameters.

Parameters	Symbol	Value	Reference
U atomic diffusion coefficient	$D_{U}$	$9.1\times 10^{-13}exp\left(\frac{-48,000\;J/mol}{RT}\right)m^{2}/s$	[30]
Zr atomic diffusion coefficient	$D_{Zr}$	$5.1\times 10^{-13}exp\left(\frac{-51,000\;J/mol}{RT}\right)m^{2}/s$	[31]
Concentration gradient coefficient	$\kappa_{c_{Zr}}$	$2.23\times 10^{-8}\;J/m$	
Phase gradient coefficient	$\kappa_{\eta }$	$1.5\times 10^{-8}\;J/m$	[32]
Phase mobility	*L*	$2.5\times 10^{-10}\;m^{3}/(Js)$	[32]
U lattice constant	$a_{U}$	0.353 nm	[33]
Zr lattice constant	$a_{Zr}$	0.357 nm	[34]

## Data Availability

The original contributions presented in this study are included in the article material, and further inquiries can be directed to the corresponding author.
